# An answered call for aid? Cannabinoid clinical framework for the opioid epidemic

**DOI:** 10.1186/s12954-023-00842-6

**Published:** 2023-08-16

**Authors:** Krista Hammaker, Nathaniel Weathington, Joseph Maroon, Lawton W. Tang, Brian Donohue, Rachel Yehuda, Kenneth M. Ford, Myro Figura, Ben Kelmendi, Belinda Tan, Matthew W. Cook, Steven D. Factor, Laura Lagano, Henry Patrick Driscoll, Adam S. Howe, EunBit G. Cho, David M. Rabin

**Affiliations:** 1 Northeast Ohio Medical University, 4209 St Rt 44, PO Box 95, Rootstown, OH 44272 USA; 2 The Board of Medicine, 1942 5th Ave, Pittsburgh, PA 15219 USA; 3 University of Pittsburgh Medical Center, 200 Delafield Rd, Ste 2040, Pittsburgh, PA 15215 USA; 4 University of Pittsburgh Medical Center, 1218 Scaife Hall, 3550 Terrace St, Pittsburgh, PA 15261 USA; 5 University of Pittsburgh Medical Center, 1300 Oxford Dr, Bethel Park, PA 15102 USA; 6 Huntington Hospital, 100 West California Blvd, Pasadena, CA 91105 USA; 7 Icahn School of Medicine at Mount Sinai, 1 Gustave L. Levy Pl, New York, NY 10029 USA; 8 Institute for Human and Machine Cognition (IHMC), 40 South Alcaniz, Pensacola, FL 32502 USA; 9 University of California Los Angeles, 757 Westwood Plaza, Ste 3325, Los Angeles, CA 90095-7403 USA; 10 Yale University, 300 George St, Ste 901, New Haven, CT 06511 USA; 11 People Science, Inc, 3870 Del Amo Blvd, Unit 507, Torrance, CA 90503 USA; 12 BioReset Medical, 3803 S Bascom Ave, Ste 203, Campbell, CA 95008 USA; 13 Abington Neurological Associates, 1151 Old York Rd, Ste 200, Abington, PA 19001 USA; 14 Albany Medical Center, 23 Hackett Blvd, MC-108, Albany, NY 12208 USA

**Keywords:** Cannabinoids, Chronic pain, Opioids

## Abstract

**Background:**

The opioid crisis continues in full force, as physicians and caregivers are desperate for resources to help patients with opioid use and chronic pain disorders find safer and more accessible non-opioid tools.

**Main body:**

The purpose of this article is to review the current state of the opioid epidemic; the shifting picture of cannabinoids; and the research, policy, and current events that make opioid risk reduction an urgent public health challenge. The provided table contains an evidence-based clinical framework for the utilization of cannabinoids to treat patients with chronic pain who are dependent on opioids, seeking alternatives to opioids, and tapering opioids.

**Conclusion:**

Based on a comprehensive review of the literature and epidemiological evidence to date, cannabinoids stand to be one of the most interesting, safe, and accessible tools available to attenuate the devastation resulting from the misuse and abuse of opioid narcotics. Considering the urgency of the opioid epidemic and broadening of cannabinoid accessibility amidst absent prescribing guidelines, the authors recommend use of this clinical framework in the contexts of both clinical research continuity and patient care.

## Background

Healthcare systems continue to struggle in the face of narcotic overuse and are largely uninformed in the use of cannabinoid-based medicines for harm-reduction in the opioid crisis. There have been many calls to explore the utility of cannabinoids for pain management and to alleviate the opioid epidemic [1, 2]. These calls emphasized the urgency of the opioid epidemic, the utility of cannabinoids, and the growing opportunities presented by increased legalization.

Through the present work, The Board of Medicine, a 501(c)3 nonprofit medical board, aims to answer these calls to action. We work to continuously evaluate all available peer-reviewed data to date on cannabinoids and other currently unregulated natural medicines. In this paper, we will review the history of the opioid epidemic and the evidence surrounding the use of cannabinoids for harm reduction in the opioid crisis. Then, considering the context of broadening legalization, absent provider recommendations, and research limitations, we provide one of the first evidence-based clinical frameworks for the utilization of cannabinoids to treat patients with chronic pain who are on opioids, seeking alternatives to opioids, and tapering opioids. It is also our hope that this framework will be helpful to standardize interventions to support much needed systematic reviews and meta-analyses of cannabinoids for pain.

## Main text

### Introduction

Despite noble intentions, many physicians feel their hands are tied when it comes to pain management and prescribing narcotics [3]. The World Health Organization (WHO) estimates that there are an annual 0.5 million worldwide deaths attributable to drug use and 70% are attributed to opioids [4]. An estimated 2 million Americans (0.7%) aged 12 years and older meet criteria for opioid use disorder [5], as we remain amid a historic shift in responsibility for the opioid epidemic, from patients to physicians to pharmaceutical companies. Purdue Pharma filed for Chapter 11 bankruptcy in 2019 [6], and it is now widely accepted that they intentionally deceived physicians and the medical community by advertising OxyContin® (oxycodone hydrochloride) as less prone to abuse than existing alternatives to increase profits [7]. In the 1960s, over 80% of people entering treatment for heroin addiction started their habit on heroin [8]. Now, about 80% of people who use heroin misused prescription opioids first [9] and most opioid overdose deaths involve synthetic opioids [10]. COVID-19 has further aggravated the opioid epidemic [11, 12], with the US reaching the grim milestone of over 100,000 overdose deaths in a 12 month period in 2021 [13]. Before COVID-19, the estimated economic burden of opioid misuse in the US was roughly $78.5 billion annually [14], but by 2022 overdoses alone were estimated to cost over $1 trillion [15].

21% of US adults experience chronic pain [16], and when opioids are used for chronic pain, evidence demonstrates that desensitization and tolerance develop quickly [17]. The need for escalating doses often causes opioid-induced hyperalgesia, making pain nearly impossible to treat, a problem that is absent with cannabinoids [18]. Further, endocannabinoid system disruptions have been linked to post-traumatic stress disorder (PTSD) [19], which is highly comorbid in patients with opioid addiction [20] and chronic pain [21], and evidence suggests cannabinoids may be therapeutic in this condition as well [22].

Despite the facts that 91% of Americans support cannabis legalization [23], there is legal medical cannabis in 38 US states [24], and cannabis is federally legal in Canada, the medical community’s utilization of cannabinoids has been limited despite a worsening opioid crisis. In February 2022, the CDC issued the agency’s first revised guidelines on opioid prescribing since 2016, which strongly urged providers to “first turn to ‘non-opioid therapies’ for both chronic and acute pain” [25]. Given the lack of educational resources and guidance around effective and minimal-risk non-opioid alternatives, this begs the question: is the medical community positioned to effectively address the changing tides of public opinion and critical public health needs when there could not be a more relevant Hippocratic sentiment to remember than “first do no harm”?

### Evidence for the utility of cannabinoids

#### Can cannabinoids alleviate the opioid crisis?

The debate over whether cannabinoids decrease opioid demand is longstanding and has compelling evidence in the US [26–31] and Canada [32–34], despite heterogeneous data that are largely an effect of legal restrictions on sourcing cannabis for research.

A 2018 longitudinal analysis showed that US prescriptions for all opioids fell by 14.4% when medical cannabis dispensaries opened, particularly for hydrocodone and morphine, but also for benzodiazepines, stimulants, and many other medications known to be over-prescribed and addictive [35]. This expeditious reduction in prescriptions was previously thought to be unachievable and the trend has extended to fentanyl-related overdose deaths [31, 34], which represent the greatest driver of the current epidemic. Questions remain about the longevity and causality of this effect after a 2014 study showed medical cannabis laws slowed increases in opioid mortality by an astonishing 24.8% [36], but a 2019 follow-up study showed trend reversal [37] and a systematic review and meta-analysis showed small non-significant reductions in opioid prescriptions and overdose mortality in states with operational marijuana dispensaries [38]. These studies highlight the importance of not using ecological correlations to draw causal conclusions, but the question of cannabis’ ability to alleviate the opioid crisis has remained open and been studied in diverse settings.

Observational research has explored the role that adult cannabis use plays for patients in pain management, substance use and mental health treatment, and harm reduction [39–41], especially among people who inject drugs (PWID) [42, 43]. This harm reduction role has also been confirmed in studies with vulnerable young people experiencing street entrenchment in Canada, who have a prevalence of cannabis use estimated as high as 98% [44, 45], with nearly 20% having sold cannabis in the past 6 months [46]. Research on patterns of use in these street-involved youth has observed associations with lower rates of initiation of injection drug use [47], the role of cannabis to be considered medicinal rather than recreational [48], harm-reduction from other more deadly substances [49], and transitioning away from more harmful forms of substance abuse [50].

Research among opioid users who are financially stable enough to frequent dispensaries has also been promising. A survey of 2897 medical cannabis patients found that, of the 34% who used opioid-based pain medication in the prior six months, 97% decreased their opioid consumption with medical cannabis and 81% said cannabis alone was more effective than cannabis plus opioids [43]. A retrospective cross-sectional survey of 1513 dispensary members indicated that 76.7% of regular opioid-using respondents reduced their use after starting medical cannabis, an effect that extended to use of alcohol (42.0%) and psychoactive medications for anxiety (71.8%), migraine (66.7%), sleep (65.2%), and depression (37.6%). When these participants were asked what they like most about cannabis, the most common response was that it helped with pain [51].

While the data supporting cannabinoids for pain are compelling, it is also conflicting and heterogeneous. For example, some data indicate that cannabis reduces self-efficacy in the frequently co-morbid conditions of depression and anxiety [52]. A four-year longitudinal observational study of cannabis use for cancer-related pain showed no opioid-sparing effect or reduction in pain severity, but only 6.5% of the 1514 patients used cannabis regularly 21–31 days per month, quality and type of cannabis was not assessed, and all patients were using black-market cannabis due to illegality during the study period [53]. For over 50 years, prospective studies of cannabis in the USA could only source plant material from one university until 2021 when the Drug Enforcement Agency (DEA) expanded access in response to quality issues [54]. This makes available data with interventional and longitudinal trials on the popular use of cannabinoids heterogeneous and extremely difficult to filter for systematic reviews and meta-analyses [55, 56].

Pure cannabinoid drugs such as oral synthetic tetrahydrocannabinol (THC) (dronabinol or Marinol®) and oral CBD (Epidiolex®) represent the most conventional prescription cannabinoid-based medicines. Dronabinol was FDA-approved in 1985 for nausea and vomiting associated with chemotherapy. In 1992, this product was approved to treat cachexia in AIDS patients [57]. In 2018 and after much public outcry, Epidiolex® was approved for the treatment of two rare seizure disorders (Lennox-Gastaut Syndrome and Dravet Syndrome) [58, 59]. Despite the growing availability of these cannabinoid-containing mainstream pharmaceuticals, there remains a significant disconnect between provider prescribing habits and the popular utilization of cannabis, which can be largely attributed to absent prescribing guidelines and unavailable standards for over-the-counter phytocannabinoid products.

#### Cannabinoid safety and utilization despite low provider knowledge

The safety of high-quality CBD has been well-established [60, 61]. Compared to most drugs for pain, psychiatric, or mood disorders, side effects of cannabis when misused are mild and can occasionally include sleepiness, diarrhea, changes in appetite/weight, cognitive effects, hyperemesis, nausea, sedation, and addiction/dependence [61, 62]. Recent research has shown a role for CBD in treating cannabis use disorder [63]. THC ingestion by children and young adults has also been associated with an increased risk of psychosis [64] and schizophrenia [65], though new research has shed light on the complexity of this correlation with shared genetic susceptibility [66]. Comparing the number needed to treat and the number needed to harm (NNH) for opiates and cannabinoids is challenging as the NNH rarely accounts for the long-term risk of opioid use disorder (OUD). However, the number needed to kill after 2.6 years of opioid therapy is 550 [67], while deaths secondary to CBD and THC have not been reported [68].

While the evidence to date suggests that cannabis is likely safe and potentially impactful, prescribers are largely uninformed about the use of cannabis-based medicines and the endocannabinoid system [69], leaving patients to self-medicate with cannabis in experimental ways [70]. A survey of 489 dispensary customers in Quebec, Canada showed that 74% had spent over one year self-medicating with cannabis, 56% were taking prescription medications, and 39% never consulted a resource on cannabis. 47% said they sometimes or never declared cannabis use to physicians and 80% said they would like to access advice from healthcare professionals about cannabis [71]. A study of 628 cannabis for therapeutic purposes (CTP) users in Canada demonstrated that barriers exist due to stigma, specifically related to patients’ perception of their providers’ attitude toward them. 48% wanted to discuss CTP with a physician but did not, and the most frequent reason (62%) for not discussing it despite a desire to do so was “don’t feel comfortable” [72]. A 2018 study in the *Journal of Clinical Oncology* shed light on critical gaps in research, education, and policy, with its survey of 400 medical oncologists that found, while 46% recommended medical cannabis and almost 80% discussed it, only 30% of oncologists felt sufficiently informed to recommend medical cannabis [69].

#### Clinical framework for prescribing cannabinoids in clinical practice and research

*The Board of Medicine* aims to address the lack of provider knowledge of cannabis, heterogenous research interventions, and untapped harm-reduction potential with an evidence-based clinical framework for utilizing cannabinoids to treat chronic pain in patients who are on opioids, seeking alternatives to opioids, and tapering opioids. The expertise of the board members was called on for review and discussion of the available evidence to generate the clinical framework found in Table 1. Board members met regularly to discuss cannabinoid dosing and opioid tapering regimens, until the final framework was agreed upon.

**Table 1:**
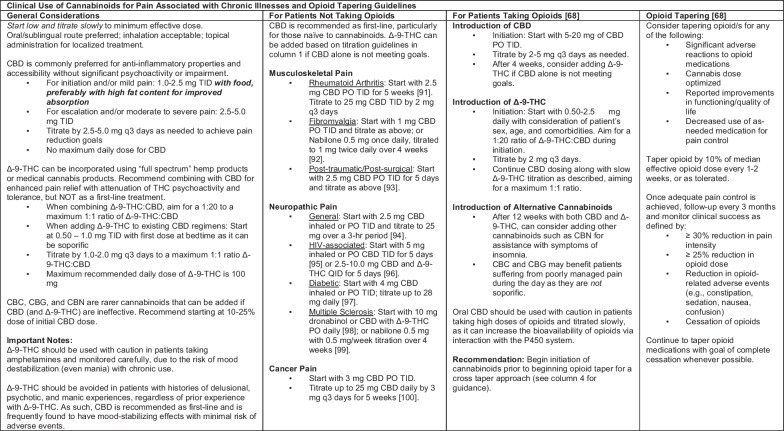
Evidence-based guidelines for the clinical use of cannabinoids for management of chronic pain in the absence of opioids, in the presence of opioids, and for those attempting to taper opioids

*Best Practices for Product Recommendation* Since CBD is derived from the hemp plant, multiple formulations are available on the market and are not subject to FDA regulation. We recommend only products that meet the following qualifications: manufactured in a clean and certified facility (ideally GMP and CO_2_ or ethanol extraction for consistency), full panel testing of the end product (for primary 8 cannabinoids and terpenes), available for consumer review, analysis verifying minimum acceptable levels of contaminants per California cannabis standards (i.e., herbicides, pesticides, fungicides), no heavy metals, no harmful solvents, no harmful or toxic filters, cannabinoid content should match label claims within a 10% variance. Considerations based on provider and patient preference include the presence of sub-cannabinoids and terpenes with maximum allowable THC content (full spectrum products are generally recommended), no synthetic or genetically engineered ingredients, and cultivation in compliance with organic farming practices. Based on current product market availability, CBD manufacturers that meet this high standard of quality and consistency for clinical use that are nationally available can be found in the following reference [101]. Guidelines and recommendations are subject to change based on new evidence

The open-access framework includes opioid tapering recommendations that are in accordance with the CDC’s newest clinical practice guidelines for managing opioids for pain. The new CDC guideline recommends the following opioids tapers: “Tapers of approximately 10% per month or slower are likely to be better tolerated than more rapid tapers when patients have been taking opioids for longer durations (e.g., ≥ 1 year). When patients have taken opioids for shorter durations (e.g., weeks to months rather than years), a decrease of 10% of the original dose per week or slower (until approximately 30% of the original dose is reached, followed by a weekly decrease of approximately 10% of the remaining dose)” [73]. We invite any clinician and researcher to access this framework to help our medical community expand our toolbox to combat the opioid epidemic and other complex public health crises with less risky non-opioid tools.

Due to the resulting low quality of available research, the International Association for the Study of Pain Presidential Task Force could not endorse the general use of cannabis and cannabinoids for pain relief [74]. Further, new CDC guidelines on prescribing opioids for pain cites the evidence base supporting cannabis for pain management as limited [73]. While these authors agree with the CDC that more research is needed to understand how to best use cannabinoids in clinical care, the gravity of the opioid epidemic, the rapid expansion of recreational cannabinoid use, and the lack of clinician education in this domain suggest an urgent need for general guidelines focused around patient safety that can be utilized in research and clinical practice, particularly for our most vulnerable patients currently dependent on opioids.

## Conclusion

### Moving forward

As opioid deaths continue to be a global problem, patients are increasingly self-medicating with cannabis [71, 75] while researchers struggle to standardize protocols and providers feel uncomfortable recommending cannabinoids amidst absent prescribing guidelines [69]. If we consider cannabis as a harm reduction tool that patients are already using without medical guidance, we can realign our focus to supporting researchers and providers with a clinical framework for standardizing research and recommending cannabinoids more informatively as safe, effective, accessible tools for assisting in the management of chronic pain. To our knowledge, this is one of the first comprehensive evidence-based peer-reviewed clinical frameworks for the safe use of cannabinoid products for chronic pain and OUD.

We believe that there is a precedent for the safe use of cannabinoids as risk-reduction tools in the clinical settings described, and that healthcare systems can integrate cannabinoids into viable treatment options as well as areas of clinical inquiry. However, there are many issues to confront. One question regards understanding the difference between phytocannabinoids compared to isolated and synthetic cannabinoids in treatment for pain. Another question regards whether the entourage effect plays a role in the action, tolerance, and side effect profile of cannabis. Most research on the cannabis plant’s molecular constituents has focused entirely on CBD and delta-9-THC, despite hundreds of known biologically active molecules present. New studies of novel phytocannabinoids suggest benefits from acidic cannabinoids [76, 77], cannabinol (CBN) [78, 79], cannabigerol (CBG) [80–87], and cannabichromene (CBC) [83, 88–90] among others.

Indeed, if phytocannabinoids prove to be non-inferior in safety and efficacy, then dissemination of this knowledge becomes a matter of social justice, because it could offer individuals of low socioeconomic status dramatically improved access to non-opioid pain management options. Furthermore, clinical adoption of cannabis-based therapies could justify insurance coverage of cannabis, which may be critical for lower income patients [72]. A foreseeable regulatory challenge regards the self-production of cannabis, and the resources patients may need (land, power, etc.) or legal obstacles they could face based on their state or regional location. Finally, until there is a federal consensus on the legality of medical cannabis, safeguards for cannabis-using patients may be limited including, for example, cannabis possession-related child endangerment laws.

Resistance to cannabis-based medicines for the opioid epidemic may have many origins, particularly the stigma associated with recreational cannabis use. That said, the evidence to date suggests that it is time for a sea change in the clinical approach to cannabis for pain management and OUD. Throughout the history of science and clinical medicine, there have been transformative changes that were initially considered heretical: hand hygiene as a means to prevent infection prior to germ theory, therapy for *H. pylori* to combat peptic ulcer disease, and even the genetic basis of cancer were all dismissed by their era’s established medical communities. Similarly, we face great resistance to the implementation of CBD and other cannabinoids for treatment-resistant chronic illnesses, despite the compelling evidence, strong overall safety profile, and urgent need. Many of our patients have already begun their own self-guided journey into pain management with cannabinoids and the burden is now on providers to consolidate the information available, conduct more rigorous research, form best practices, and implement guidelines that will inform both the field and those we care for without stigma.

“To study the phenomena of disease without books is to sail an uncharted sea, while to study books without patients is not to go to sea at all.”—William Osler

## Data Availability

All data are available in the main text or the supplementary materials.

## Abbreviations

CBD: Cannabidiol
CDC: Centers for Disease Control and Prevention
PTSD: Post-traumatic stress disorder
PCPs: Primary care physicians
PWID: People who inject drugs
THC: Tetrahydrocannabinol
OUD: Opioid use disorder
NNH: Number needed to harm

## References

[1] Hurd YL (2017). Cannabidiol: swinging the marijuana pendulum from 'weed' to medication to treat the opioid epidemic. Trends Neurosci.

[2] Lucas P (2017). Rationale for cannabis-based interventions in the opioid overdose crisis. Harm Reduct J.

[3] Desveaux L, Saragosa M, Kithulegoda N, Ivers NM (2019). Understanding the behavioural determinants of opioid prescribing among family physicians: a qualitative study. BMC Fam Pract.

[4] Organization WH. Opioid Overdose: WHO Newsroom 2021. https://www.who.int/news-room/fact-sheets/detail/opioid-overdose.

[5] Centers for Disease Control and Prevention. 2019 annual surveillance report of drug-related risks and outcomes—United States surveillance special report 2019 [June 6, 2022]. https://www.cdc.gov/drugoverdose/pdf/pubs/2019-cdc-drug-surveillance­report.pdf.

[6] Hoffman J, Walsh M. Purdue Pharma, maker of OxyContin, files for bankruptcy The New York Times The New York Times; 2020. https://www.nytimes.com/2019/09/15/health/purdue-pharma-bankruptcy-opioids-settlement.html.

[7] Meier B. In guilty plea, OxyContin maker to pay $600 million: The New York Times 2007 [updated May 10, 2007]. https://www.nytimes.com/2007/05/10/business/11drug-web.html.

[8] Cicero TJ, Ellis MS, Surratt HL, Kurtz SP (2014). The changing face of heroin use in the United States: a retrospective analysis of the past 50 years. JAMA Psychiatry.

[9] Muhuri P, Gfroerer J, Davies M. Associations of nonmedical pain reliever use and initiation of heroin use in the United States Center for Behavioral Health Statistics and Quality: Substance Abuse and Mental Health Services Administration; 2013. https://www.samhsa.gov/data/sites/default/files/DR006/DR006/nonmedical-pain-reliever-use-2013.pdf.

[10] Julie K, O’Donnell P, John Halpin M, Christine L, Mattson P, Bruce A, Goldberger P, Gladden RM (2017). Deaths involving fentanyl, fentanyl analogs, U-47700—10 states, July-December 2016. Morb Mortal Wkly Rep.

[11] Ochalek TA, Cumpston KL, Wills BK, Gal TS, Moeller FG (2020). Nonfatal opioid overdoses at an urban emergency department during the COVID-19 pandemic. JAMA.

[12] Niles JK, Gudin J, Radcliff J, Kaufman HW (2021). The opioid epidemic within the COVID-19 pandemic: drug testing in 2020. Popul Health Manag.

[13] Centers for Disease Control and Prevention. Drug overdose deaths in the U.S. top 100,000 annually National Center for Health Statistics2021. https://www.cdc.gov/nchs/pressroom/nchs_press_releases/2021/20211117.htm.

[14] Florence CS, Zhou C, Luo F, Xu L (2016). The economic burden of prescription opioid overdose, abuse, and dependence in the United States, 2013. Med Care.

[15] Trafficking CoCSO. Commission on combating synthetic opioid traffiking RAND corporations 2022. https://www.rand.org/pubs/external_publications/EP68838.html.

[16] Nahin RL, Feinberg T, Kapos FP, Terman GW (2023). Estimated rates of incident and persistent chronic pain among US adults, 2019–2020. JAMA Netw Open.

[17] Dowell D, Haegerich TM, Chou R (2016). CDC guideline for prescribing opioids for chronic pain-United States, 2016. JAMA.

[18] St Pierre M, Russo EB, Walsh Z (2020). No evidence of altered reactivity to experimentally induced pain among regular cannabis users. Clin J Pain.

[19] Hill MN, Campolongo P, Yehuda R, Patel S (2018). Integrating endocannabinoid signaling and cannabinoids into the biology and treatment of posttraumatic stress disorder. Neuropsychopharmacology.

[20] Bilevicius E, Sommer JL, Asmundson GJG, El-Gabalawy R (2018). Posttraumatic stress disorder and chronic pain are associated with opioid use disorder: results from a 2012–2013 American nationally representative survey. Drug Alcohol Depend.

[21] Kind S, Otis JD (2019). The interaction between chronic pain and PTSD. Curr Pain Headache Rep.

[22] Orsolini L, Chiappini S, Volpe U, Berardis D, Latini R, Papanti GD (2019). Use of medicinal cannabis and synthetic cannabinoids in post-traumatic stress disorder (PTSD): a systematic review. Medicina (Kaunas).

[23] Green TV. Americans overwhelmingly say marijuana should be legal for recreational or medical use: Pew Research Center 2021. https://www.pewresearch.org/fact-tank/2021/04/16/americans-overwhelmingly-say-marijuana-should-be-legal-for-recreational-or-medical-use/.

[24] Medical Marijuana Laws: NORML; 2022. https://norml.org/laws/medical-laws/.

[25] Hoffman J. CDC proposes new guidelines for treating pain, including opioid use The New York Times 2022 [updated February 10, 2022. https://www.nytimes.com/2022/02/10/health/cdc-opioid-pain-guidelines.html.

[26] Wen H, Hockenberry JM (2018). Association of medical and adult-use marijuana laws with opioid prescribing for medicaid enrollees. JAMA Intern Med.

[27] Ishida JH, Wong PO, Cohen BE, Vali M, Steigerwald S, Keyhani S (2019). Substitution of marijuana for opioids in a national survey of US adults. PLoS ONE.

[28] Shah A, Hayes CJ, Lakkad M, Martin BC (2019). Impact of medical marijuana legalization on opioid use, chronic opioid use, and high-risk opioid use. J Gen Intern Med.

[29] Bradford A, Bradford D (2016). Medical marijuana laws reduce prescription medication use in medicare part D. Health Aff (Millwood).

[30] Liang D, Bao Y, Wallace M, Grant I, Shi Y (2018). Medical cannabis legalization and opioid prescriptions: evidence on US Medicaid enrollees during 1993–2014. Addiction.

[31] Hsu G, Kovacs B (2021). Association between county level cannabis dispensary counts and opioid related mortality rates in the United States: panel data study. BMJ.

[32] Dranitsaris G, DeAngelis C, Pearson B, McDermott L, Pohlmann-Eden B (2021). Opioid prescribing in Canada following the legalization of cannabis: a clinical and economic time-series analysis. Appl Health Econ Health Policy.

[33] Meng H, Page MG, Ajrawat P, Deshpande A, Samman B, Dominicis M (2021). Patient-reported outcomes in those consuming medical cannabis: a prospective longitudinal observational study in chronic pain patients. Can J Anaesth.

[34] Socias ME, Choi J, Lake S, Wood E, Valleriani J, Hayashi K (2021). Cannabis use is associated with reduced risk of exposure to fentanyl among people on opioid agonist therapy during a community-wide overdose crisis. Drug Alcohol Depend.

[35] Bradford AC, Bradford WD, Abraham A, Bagwell AG (2018). Association between US State medical cannabis laws and opioid prescribing in the medicare part D population. JAMA Intern Med.

[36] Bachhuber MA, Saloner B, Cunningham CO, Barry CL (2014). Medical cannabis laws and opioid analgesic overdose mortality in the United States, 1999–2010. JAMA Intern Med.

[37] Shover CL, Davis CS, Gordon SC, Humphreys K (2019). Association between medical cannabis laws and opioid overdose mortality has reversed over time. Proc Natl Acad Sci U S A.

[38] Chihuri S, Li G (2019). State marijuana laws and opioid overdose mortality. Inj Epidemiol.

[39] Lau N, Sales P, Averill S, Murphy F, Sato SO, Murphy S (2015). A safer alternative: cannabis substitution as harm reduction. Drug Alcohol Rev.

[40] Lucas P, Walsh Z, Crosby K, Callaway R, Belle-Isle L, Kay R (2016). Substituting cannabis for prescription drugs, alcohol and other substances among medical cannabis patients: the impact of contextual factors. Drug Alcohol Rev.

[41] Hill KP (2015). Medical marijuana for treatment of chronic pain and other medical and psychiatric problems: a clinical review. JAMA.

[42] Kral AH, Wenger L, Novak SP, Chu D, Corsi KF, Coffa D (2015). Is cannabis use associated with less opioid use among people who inject drugs?. Drug Alcohol Depend.

[43] Reiman A, Welty M, Solomon P (2017). Cannabis as a substitute for opioid-based pain medication: patient self-report. Cannabis Cannabinoid Res.

[44] Saddichha S, Linden I, Krausz MR (2014). Physical and mental health issues among homeless youth in British Columbia, Canada: are they different from older homeless adults?. J Can Acad Child Adolesc Psychiatry.

[45] Werb D, Kerr T, Buxton J, Shoveller J, Richardson C, Montaner J (2013). Crystal methamphetamine and initiation of injection drug use among street-involved youth in a Canadian setting. CMAJ.

[46] Reddon H, Fast D, DeBeck K, Werb D, Hayashi K, Wood E (2019). Prevalence and correlates of selling illicit cannabis among people who use drugs in Vancouver, Canada: a ten-year prospective cohort study. Int J Drug Policy.

[47] Reddon H, DeBeck K, Socias ME, Dong H, Wood E, Montaner J (2018). Cannabis use is associated with lower rates of initiation of injection drug use among street-involved youth: a longitudinal analysis. Drug Alcohol Rev.

[48] Paul B, Thulien M, Knight R, Milloy MJ, Howard B, Nelson S (2020). "Something that actually works": cannabis use among young people in the context of street entrenchment. PLoS ONE.

[49] Bozinoff N, Small W, Long C, DeBeck K, Fast D (2017). Still, "at risk": an examination of how street-involved young people understand, experience, and engage with "harm reduction" in Vancouver's inner city. Int J Drug Policy.

[50] Boyd J, Fast D, Hobbins M, McNeil R, Small W (2017). Social-structural factors influencing periods of injection cessation among marginalized youth who inject drugs in Vancouver, Canada: an ethno-epidemiological study. Harm Reduct J.

[51] Piper B, DeKeuster R, Beals ML, Cobb CM, Burchman CA, Perkinson L (2017). Substitution of medical cannabis for pharmaceutical agents for pain, anxiety and sleep. J Psychopharmacol.

[52] Wilson M, Gogulski HY, Cuttler C, Bigand TL, Oluwoye O, Barbosa-Leiker C (2018). Cannabis use moderates the relationship between pain and negative affect in adults with opioid use disorder. Addict Behav.

[53] Campbell G, Hall WD, Peacock A, Lintzeris N, Bruno R, Larance B (2018). Effect of cannabis use in people with chronic non-cancer pain prescribed opioids: findings from a 4-year prospective cohort study. Lancet Public Health.

[54] Zagorski N. Experts reflect on what new law might mean for cannabis research Psychiatry Online American Psychiatric Association; 2023.

[55] Gedin F, Blome S, Ponten M, Lalouni M, Fust J, Raquette A (2022). Placebo response and media attention in randomized clinical trials assessing cannabis-based therapies for pain: a systematic review and meta-analysis. JAMA Netw Open.

[56] Lake S, St PM (2020). The relationship between cannabis use and patient outcomes in medication-based treatment of opioid use disorder: a systematic review. Clin Psychol Rev.

[57] Watson SJ, Benson JA, Joy JE (2000). Marijuana and medicine: assessing the science base: a summary of the 1999 Institute of Medicine report. Arch Gen Psychiatry.

[58] Kelley K. FDA approves first marijuana-derived drug NEJM Journal Watch; 2018. https://www.jwatch.org/fw114313/2018/06/26/fda-approves-first-marijuana-derived-drug. 10.1038/nrd.2018.131 30057413

[59] FDA approves new indication for drug containing an active ingredient derived from cannabis to treat seizures in rare genetic disease [press release]. FDA News Release, July 31, 2020.

[60] Iffland K, Grotenhermen F (2017). An update on safety and side effects of cannabidiol: a review of clinical data and relevant animal studies. Cannabis Cannabinoid Res.

[61] Bergamaschi MM, Queiroz RH, Zuardi AW, Crippa JA (2011). Safety and side effects of cannabidiol, a *Cannabis sativa* constituent. Curr Drug Saf.

[62] McDonagh MS, Selph SS, Buckley DI, Holmes RS, Mauer K, Ramirez S, et al. Nonopioid pharmacologic treatments for chronic pain. AHRQ Comparative Effectiveness Reviews. Rockville (MD) 2020. 32338847

[63] Freeman TP, Hindocha C, Baio G, Shaban NDC, Thomas EM, Astbury D (2020). Cannabidiol for the treatment of cannabis use disorder: a phase 2a, double-blind, placebo-controlled, randomised, adaptive Bayesian trial. Lancet Psychiatry.

[64] Marconi A, Di Forti M, Lewis CM, Murray RM, Vassos E (2016). Meta-analysis of the association between the level of cannabis use and risk of psychosis. Schizophr Bull.

[65] Godin SL, Shehata S (2022). Adolescent cannabis use and later development of schizophrenia: an updated systematic review of longitudinal studies. J Clin Psychol.

[66] Cheng W, Parker N, Karadag N, Koch E, Hindley G, Icick R (2023). The relationship between cannabis use, schizophrenia, and bipolar disorder: a genetically informed study. Lancet Psychiatry.

[67] Frieden TR, Houry D (2016). Reducing the risks of relief-the CDC opioid-prescribing guideline. N Engl J Med.

[68] Sihota A, Smith BK, Ahmed SA, Bell A, Blain A, Clarke H (2021). Consensus-based recommendations for titrating cannabinoids and tapering opioids for chronic pain control. Int J Clin Pract.

[69] Braun IM, Wright A, Peteet J, Meyer FL, Yuppa DP, Bolcic-Jankovic D (2018). Medical oncologists' beliefs, practices, and knowledge regarding marijuana used therapeutically: a nationally representative survey study. J Clin Oncol.

[70] DiGrande S (2018). Medical marijuana in cancer treatment: no standards of care, and so far, no coverage. Am J Manag Care.

[71] Asselin A, Lamarre OB, Chamberland R, McNeil SJ, Demers E, Zongo A (2022). A description of self-medication with cannabis among adults with legal access to cannabis in Quebec, Canada. J Cannabis Res.

[72] Belle-Isle L, Walsh Z, Callaway R, Lucas P, Capler R, Kay R (2014). Barriers to access for Canadians who use cannabis for therapeutic purposes. Int J Drug Policy.

[73] Dowell D, Ragan KR, Jones CM, Baldwin GT, Chou R (2022). CDC clinical practice guideline for prescribing opioids for pain: United States, 2022. MMWR Recomm Rep.

[74] Haroutounian S, Arendt-Nielsen L, Belton J, Blyth FM, Degenhardt L, Di Forti M (2021). International association for the study of pain presidential task force on cannabis and cannabinoid analgesia: research agenda on the use of cannabinoids, cannabis, and cannabis-based medicines for pain management. Pain.

[75] Erridge S, Coomber R, Sodergren MH (2022). Medical cannabis, CBD wellness products and public awareness of evolving regulations in the United Kingdom. J Cannabis Res.

[76] Nguyen LC, Yang D, Nicolaescu V, Best TJ, Gula H, Saxena D (2022). Cannabidiol inhibits SARS-CoV-2 replication through induction of the host ER stress and innate immune responses. Sci Adv..

[77] van Breemen RB, Muchiri RN, Bates TA, Weinstein JB, Leier HC, Farley S (2022). Cannabinoids block cellular entry of SARS-CoV-2 and the emerging variants. J Nat Prod.

[78] Lavender I, McGregor IS, Suraev A, Grunstein RR, Hoyos CM (2022). Cannabinoids, insomnia, and other sleep disorders. Chest.

[79] Corroon J (2021). Cannabinol and sleep: separating fact from fiction. Cannabis Cannabinoid Res.

[80] Cascio MG, Gauson LA, Stevenson LA, Ross RA, Pertwee RG (2010). Evidence that the plant cannabinoid cannabigerol is a highly potent alpha2-adrenoceptor agonist and moderately potent 5HT1A receptor antagonist. Br J Pharmacol.

[81] Rock EM, Goodwin JM, Limebeer CL, Breuer A, Pertwee RG, Mechoulam R (2011). Interaction between non-psychotropic cannabinoids in marihuana: effect of cannabigerol (CBG) on the anti-nausea or anti-emetic effects of cannabidiol (CBD) in rats and shrews. Psychopharmacology.

[82] Deiana S, Watanabe A, Yamasaki Y, Amada N, Arthur M, Fleming S (2012). Plasma and brain pharmacokinetic profile of cannabidiol (CBD), cannabidivarine (CBDV), Delta(9)-tetrahydrocannabivarin (THCV) and cannabigerol (CBG) in rats and mice following oral and intraperitoneal administration and CBD action on obsessive-compulsive behaviour. Psychopharmacology.

[83] Stone NL, Murphy AJ, England TJ, O'Sullivan SE (2020). A systematic review of minor phytocannabinoids with promising neuroprotective potential. Br J Pharmacol.

[84] Zhou C, Assareh N, Arnold JC (2021). The cannabis constituent cannabigerol does not disrupt fear memory processes or stress-induced anxiety in mice. Cannabis Cannabinoid Res.

[85] Zagzoog A, Mohamed KA, Kim HJJ, Kim ED, Frank CS, Black T (2020). In vitro and in vivo pharmacological activity of minor cannabinoids isolated from *Cannabis sativa*. Sci Rep.

[86] Navarro G, Varani K, Reyes-Resina I, Sanchez de Medina V, Rivas-Santisteban R, Sanchez-Carnerero Callado C (2018). Cannabigerol action at cannabinoid CB1 and CB2 receptors and at CB1–CB2 heteroreceptor complexes. Front Pharmacol.

[87] Walsh KB, McKinney AE, Holmes AE (2021). Minor cannabinoids: biosynthesis, molecular pharmacology and potential therapeutic uses. Front Pharmacol.

[88] Filipiuc LE, Ababei DC, Alexa-Stratulat T, Pricope CV, Bild V, Stefanescu R (2021). Major phytocannabinoids and their related compounds: should we only search for drugs that act on cannabinoid receptors?. Pharmaceutics.

[89] Karschner EL, Swortwood-Gates MJ, Huestis MA (2020). Identifying and quantifying cannabinoids in biological matrices in the medical and legal cannabis era. Clin Chem.

[90] Russo EB (2011). Taming THC: potential cannabis synergy and phytocannabinoid-terpenoid entourage effects. Br J Pharmacol.

[91] Blake DR, Robson P, Ho M, Jubb RW, McCabe CS (2006). Preliminary assessment of the efficacy, tolerability and safety of a cannabis-based medicine (Sativex) in the treatment of pain caused by rheumatoid arthritis. Rheumatology (Oxford).

[92] Skrabek RQ, Galimova L, Ethans K, Perry D (2008). Nabilone for the treatment of pain in fibromyalgia. J Pain.

[93] Ware MA, Wang T, Shapiro S, Robinson A, Ducruet T, Huynh T (2010). Smoked cannabis for chronic neuropathic pain: a randomized controlled trial. CMAJ.

[94] Wilsey B, Marcotte T, Deutsch R, Gouaux B, Sakai S, Donaghe H (2013). Low-dose vaporized cannabis significantly improves neuropathic pain. J Pain.

[95] Abrams DI, Jay CA, Shade SB, Vizoso H, Reda H, Press S (2007). Cannabis in painful HIV-associated sensory neuropathy: a randomized placebo-controlled trial. Neurology.

[96] Ellis RJ, Toperoff W, Vaida F, van den Brande G, Gonzales J, Gouaux B (2009). Smoked medicinal cannabis for neuropathic pain in HIV: a randomized, crossover clinical trial. Neuropsychopharmacology.

[97] Wallace MS, Marcotte TD, Umlauf A, Gouaux B, Atkinson JH (2015). Efficacy of inhaled cannabis on painful diabetic neuropathy. J Pain.

[98] Svendsen KB, Jensen TS, Bach FW (2004). Does the cannabinoid dronabinol reduce central pain in multiple sclerosis? Randomised double blind placebo controlled crossover trial. BMJ.

[99] Rog DJ, Nurmikko TJ, Friede T, Young CA (2005). Randomized, controlled trial of cannabis-based medicine in central pain in multiple sclerosis. Neurology.

[100] Portenoy RK, Ganae-Motan ED, Allende S, Yanagihara R, Shaiova L, Weinstein S (2012). Nabiximols for opioid-treated cancer patients with poorly-controlled chronic pain: a randomized, placebo-controlled, graded-dose trial. J Pain.

[101] The Board of Medicine. https://www.boardofmedicine.org.

